# Management of tumor rupture and abdominal compartment syndrome in an infant with bilateral high risk stage 4 neuroblastoma: A case report

**DOI:** 10.1097/MD.0000000000016752

**Published:** 2019-08-23

**Authors:** Holger N. Lode, Günter Henze, Nikolai Siebert, Karoline Ehlert, Winfried Barthlen

**Affiliations:** a University Medicine Greifswald, Department of Pediatric Hematology and Oncology; b Clinic of Pediatric Surgery, Ferdinand-Sauerbruch-Strasse 1Greifswald, Germany.

**Keywords:** Abdominal compartment, bleeding, neuroblastoma, tumor rupture

## Abstract

**Rationale:**

Tumor rupture and bleeding at initial presentation of infants with neuroblastoma (NBL) is a rare, but life threatening condition and challenge in pediatric oncology. Here, we report successful multidisciplinary management of an abdominal compartment syndrome as a result of tumor rupture and bleeding in an infant with bilateral high risk stage 4 NBL.

**Patient concerns:**

The patient was admitted to a cooperating hospital with vomiting, failure to thrive and a large mass in the abdomen and was then referred to our center.

**Diagnoses:**

Stage 4 NBL with MYC-N amplification and 1p36 deletion was diagnosed in an 11 months old girl. Due to rapid and massive tumor growth she developed abdominal compression with renal failure, severe bleeding, and tumor lysis syndrome (TLS).

**Interventions:**

Surgical decompression by enterostomy, local, and systemic bleeding control with platelets and coagulation factors, antiinfective and TLS therapy were effective in stabilizing the patient's condition. This allowed initiation of the multimodal antineoplastic treatment according to protocol NB 2004.

**Outcomes:**

Mechanical ventilation was stopped after 11 days, the abdominal wall was closed 3 months after the start of therapy, and treatment according to the protocol be started and successfully completed.

**Lessons:**

Only the immediate, coordinated multidisciplinary intervention managed to overcome the life-threatening abdominal compartment syndrome and its associated problems, eventually enabling successful curative treatment.

## 1 Introduction

Neuroblastoma (NB) is the most common malignant solid tumors of infancy and childhood. The overall survival rate of stage 4 patients still remains poor despite intensive multimodal treatment regimens.^[1]^ In 40% to 50% of patients NB arise in the adrenal glands.^[2]^ Spontaneous abdominal hemorrhage within the adrenal glands may be a leading sign for NB and occurs particular during the neonatal period,^[3]^ less frequently in older children.^[4]^ Massive intraabdominal hemorrhage resulting in an abdominal compartment syndrome due to tumor rupture is rare and life-threatening.^[5]^ Here we report on an infant with bilateral high-risk stage 4 neuroblastoma (1p36 deletion, MYC-N amplification) presenting with tumor rupture and abdominal hemorrhage. Only by immediate, coordinated interdisciplinary action the life-threatening abdominal compartment syndrome and its associated problems could successfully be managed, eventually enabling curative treatment.

## 2 Case

**Figure 1:**
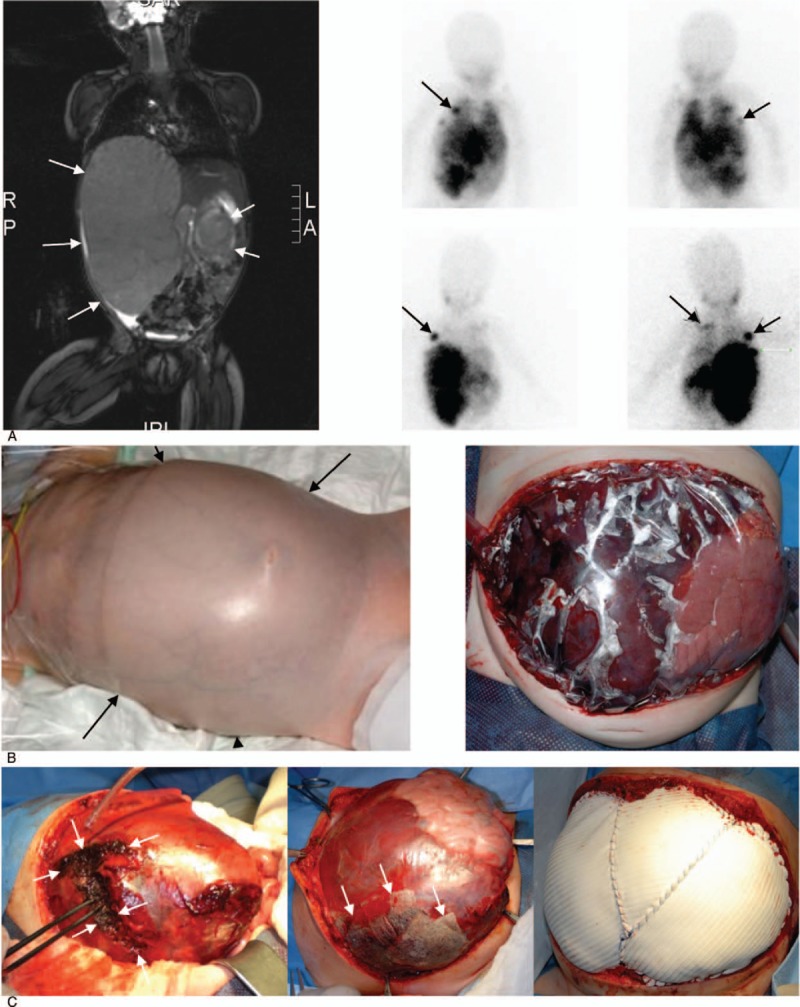
Staging and surgical treatment of an abdominal compartment syndrome in an infant (<12 months) with high risk stage 4 neuroblastoma. (A) Magnetic resonance imaging (left) and ^123^I meta-iodo benzylguanidine scintigraphy (right) illustrate the extent of the bilateral neuroblastoma at initial presentation and indicate bone metastasis. White arrows delineate left and right primary tumor manifestations on MRI, black arrows indicate bone metastases on mIBG scans (right 4th rib, left scapula). (B) Visual impression of the abdomen characterized by bluish discoloration of the skin (left). Increase gastric pressure (17 cmH_2_o), free abdominal fluid on ultrasound, compression of the abdominal vessels and decreased urinary output (<0.5 ml/kg/h) are consistent with the diagnosis of an abdominal compartment syndrome. Pressure release was accomplished after enterostomy and the right sided tumor presented with bleeding from the area of massive infiltration into right hepatic lobe. Temporary closure was accomplished with plastic drape (right). (C) In parallel to mass transfusion, application of coagulation factors, start of chemo- and radiotherapy, bleeding from the ruptured right sided intrahepatic tumor component was electrocoagulated and covered with a fibrinogen containing haemostat (TachoSil). White arrows indicate the ruptured tumor (left) and the edges of the TachoSil patches (middle). After 6 days, bleeding stopped completely and the enterostomy was closed with a gore-tex patch (right).

Parents provided written consent to report the case of their child. A Caucasian girl (11 months) was admitted to a local hospital with vomiting, failure to thrive (development of body weight, age, weight, percentile: 10 days, 3320 g, P50; 1 month, 4050 g, P50; 3 months, 5090 g, P25; 6 months, 5970 g, P3; 11 months, 6320 g, <<P3) and loss of milestones (stopped walking). Physical examination revealed a solid resistance in the right upper abdomen, and 2 massive bilateral solid tumors were diagnosed by ultrasound. Initial laboratory findings were as follows: Hemoglobin 7.8 g/dl, serum lactate dehydrogenase 3250 IU/L (<300 IU/L), uric acid 8.3 mg/dl (<5.0 mg/dl), neuron specific enolase 1084 μg/l (<16 μg/L), vanillylmandelic acid/creatinine 50 mmol/mmol (<10.7 mmol/mmol), homovanillylmandelic acid 75 mmol/mmol (<20.1 mmol/mmol). Bone marrow infiltration was not detectable by microscopy and anti-GD_2_ immunocytochemistry (aspirates and biopsies from 4 locations; 2× tibia, 2× iliac crest). Magnetic resonance imaging (MRI) and scintigraphy with ^123^I meta-iodo-benzylguanidine revealed bilateral NB (Fig. 1A) originating from both adrenal glands with metastases to retroperitoneal lymph nodes and lesions of the skeleton at the left scapula and the right 4th rib.

During staging procedures, the child developed progressive tachypnea (>40/minute), tachycardia (>150 bpm), and a distended abdomen with bluish discoloration of the skin (Fig. 1B). At the same time urine output decreased (<0.5 ml/kg/hour), and a gradual increase of the intragastric pressure (max. 17 cmH_2_O) established the diagnosis of an abdominal compartment syndrome (ACS). On ultrasound, the abdominal blood vessels (*vena cava*, abdominal aorta) were compressed, and free abdominal fluid was detectable. Based on the deteriorating clinical condition, the sustained rise of the intraabdominal pressure (IAP) together with the newly developed organ dysfunction it was decided to perform an enterostomy in order to decompress the abdominal cavity Steinau^[6]^ (Fig. 1B). Laparotomy revealed a tumor rupture with extensive bleeding located at the right abdominal component of the primary tumor (Fig. 1C) which infiltrated the entire right hepatic lobe. A tumor biopsy was taken from the smaller left sided primary tumor, and a central line was implanted (dual lumen 6,6fr broviac catheter). Closure of the abdominal cavity was not possible due to the huge ruptured tumor infiltrating the liver.

**Figure 2:**
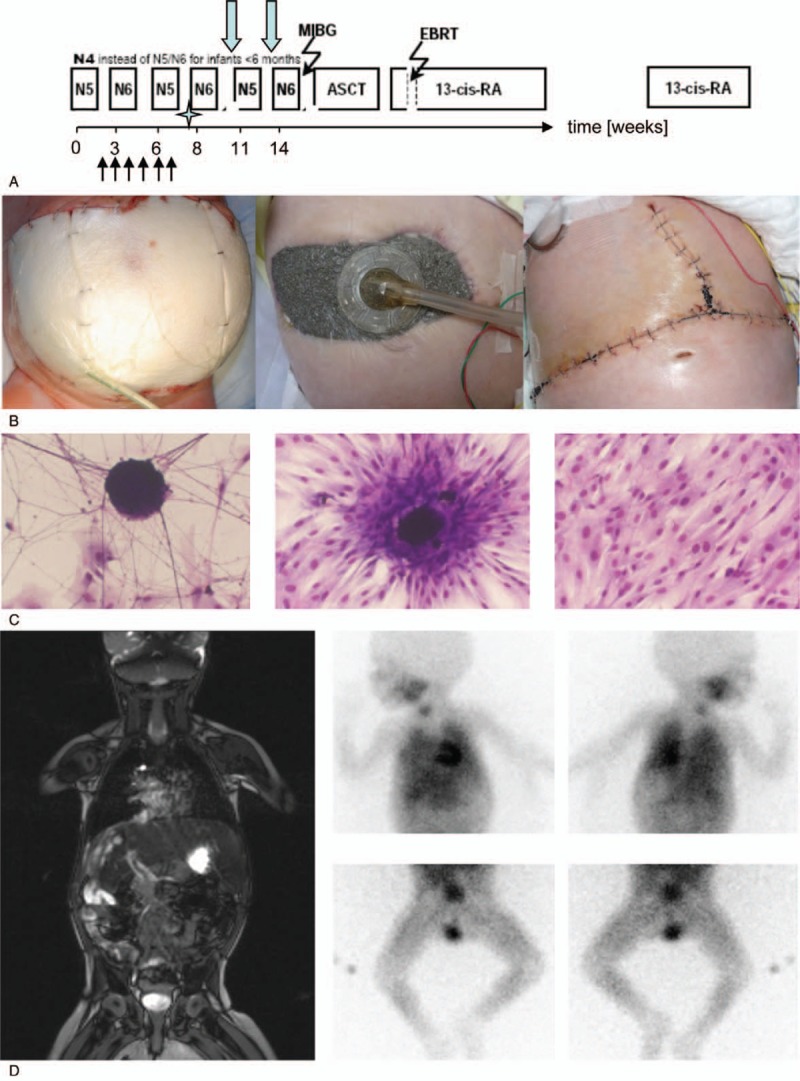
Treatment schematic and follow up after initial management of the abdominal compartment syndrome. (A) The treatment was initiated according to the current German high risk neuroblastoma protocol (NB2004). After 3 cycles, peripheral blood stem cell harvest (4 × 10^6^ CD34 /kg) was accomplished (asterisk) in order to prepare for **A**utologous **S**tem **C**ell Transplantation. Black arrows indicate time points of sequential reduction of the gore-tex patch, green arrows show time points of the surgical removal of the left and the right primary tumors. (B) After closure of the enterostomy with the gore-tex patch, a vacuum dressing was applied in order to clear the wound from secretion (left). Size reduction was accomplished as a result of cytotoxic therapy over time (middle). Closure of the abdomen was realized after removal of the primary tumors (right). (C) Small fragments of the primary tumor biopsy taken from the left sided primary neuroblastoma were cultured in RPMI yielding a stable and rapidly growing cell line. Characterization of the cell line revealed expression of ganglioside GD_2_ and MYC-N, which are typical features of neuroblastoma cells. (D) Restaging after surgical treatment and 6 cycles of chemotherapy according to the German NB2004 protocol by MRI (left) and mIBG (right) indicate a very good partial remisson (VGPR).

Small fragments of the biopsy were cultured in RPMI containing 10% human serum in a standard incubator (5% CO2, 100% H_2_O saturation) yielding a stable and rapidly growing cell line. The cells expressed ganglioside GD_2_ and were MYC-N amplified, which are typical features of neuroblastoma cells (Fig. 2C). Histology of the tumor revealed an undifferentiated stroma-poor NB Sano^[7]^ with MYC-N amplification (20–40 copies) and 1p36 deletion establishing the diagnosis of a high risk stage 4 NB Monclair^[8]^ in an infant less than twelve months of age.

During the first postoperative days the bleeding of the ruptured right sided tumor continued (Fig. 1C) requiring immediate interdisciplinary action.

First, hematologists and blood bank were required to substitute blood loss by continuous infusion of red blood cells (40 ml/hour) to maintain hemoglobin levels above 8 g/dl, fresh frozen plasma (10 ml/kg q8 hour) to keep fibrinogen above 1 g/L and platelets to keep thrombocytes above 50,000/μl with focus on homeostasis of electrolytes, blood glucose, and fluid balance. In addition, coagulation factors had to be substituted (activated factor VII, prothrombin complex, factor XIII, anti-thrombin III, and, at the same time fibrinolysis was inhibited with tranexamic acid.

In parallel, pediatric surgeons performed surgery to control bleeding in ruptured tumor areas through electrocoagulation and the use of TachoSil, a topical fibrin-based hemostatic agent (Fig. 1C).

Chemotherapy according to the German neuroblastoma protocol (NB2004) was started by pediatric oncologists, at first with chemotherapy (element N5) consisting of vindesin (0.1 mg/kg, day 1), cisplatinum (1.3 mg/kg, day 1–4) and etoposide 4.2 mg/kg, day 1–4) followed by GCSF support (Fig. 2A).

At the same time, radiotherapists began irradiating the right-sided primary tumor at low dose (5 × 1 Gy) to accelerate and assist cytoreductive therapy, thereby reducing intratumoral pressure.

In parallel, the infection team delivered a preventive antiinfective therapy with broad spectrum antibiotics and antimycotics. During the entire course of the treatment no evidence of bacterial, fungal, or viral infection was found.

Tumor lysis syndrome with hyperuricaemia was treated with fluids (3l/m^2^ body surface area), electrolytes, and furosemide in combination with rasburicase.

This multidisciplinary management was finally effective and successful. After 6 days, the bleeding was stopped, and the enterostomy could be closed by inserting a gore-tex patch accompanied by vacuum dressing (Fig. 1C, 2B). Intensive care management also included cardiovascular therapy (noradrenaline and dobutamine) and thyroxine against low T_3_ syndrome. Respirator therapy was discontinued after 11 days and the tumor lysis syndrome resolved gradually over a period of 2 weeks.

Chemotherapy was continued according to protocol NB2004 for high risk stage 4 neuroblastoma patients (Fig. 2A) with alternating N5/N6 chemotherapy cycles (N5 see above, N6: vincristine 0.05 mg/kg day 1 and 8, dacarbacine 6.7 mg/kg day 1–5, ifosfamide 50 mg/kg day 1–5, adriamycin 1 mg/kg/day 6 and 7). Between the courses peripheral blood stem cells were collected for the planned high-dose chemotherapy. Surgically, 6 successive reductions of the gore-tex patch were performed in weekly intervals followed by sequential resections of the left and right primary tumor. Finally, 3 months after the start of therapy the abdominal wall could be closed (Fig. 2B). Magnetic resonance imaging and mIBG scans indicated a very good partial remission (VGPR) (Fig. 2D). The little girl was in a good general condition, on oral feeding and gradually gaining weight and could be prepared for high dose chemotherapy followed by autologous stem cell rescue.

## 3 Discussion

The typical manifestation of NB in newborns and infants is stage 4s which is associated with a favorable outcome. The outcome related to survival of the neuroblastoma remains favorable even if laparostomy has been required in patients with rapidly growing tumors due to liver metastases resulting in an abdominal compartment syndrome (ACS).^[9,10]^ Nevertheless, mortality from ACS ranges from 50% to 60%^[11]^, and early decompression by enterostomy has been described to improve outcome.^[12]^ Another specific and in part lethal problem in newborns and young infants with NB may be disseminated intravascular coagulation.^[13,14]^

In contrast, true high-risk metastatic NB with MYC-N amplification, 1p36 deletion, and unfavorable histology in infants is rare. The aggressiveness of the NB of patient reported here is also documented by the rapid growth of tumor cells in vitro. With these cells, a new neuroblastoma cell line (HGW-CM) could be established expressing ganglioside GD_2_ and sharing *myc-n* amplification as well as 1p36 deletion like the parental tumor.

In a report from the Neuroblastoma Group of the International Society of Paediatric Oncology Europe (SIOPEN) MYC-N amplified NB accounted for only 10% of affected infants.^[15]^ Here, infants with MYC-N amplified NB had a poor outcome despite intensified therapy with a 2-year overall survival of 30% (SE, 0.08) only and a median survival time of 12 months with deaths due to disease being the most frequent events.

Published case reports are scarce on infants with high-risk NB and abdominal compartment syndrome. The patient reported here was complex with 4 life threatening conditions including high-risk NB, tumor rupture, and abdominal compartment syndrome followed by tumor lysis syndrome and severe bleeding problems.

Immediate interdisciplinary well coordinated parallel interventions of a variety of pediatric subspecialties including pediatric surgery, pediatric oncology, radiotherapy, transfusion medicine, infectiology, and supportive therapy was key to successful management of this complex condition.

Very important was an early decompression to reinstitute renal function as it is essential for the timely clearance of cytotoxic agents as well as for the management of the tumor lysis syndrome. It is speculative, which further intervention was most important, but the outcome demonstrates impressively that such conditions require the full commitment of all disciplines involved.

In conclusion, timely enterostomy is capable of effectively bridging a life threatening abdominal compartment syndrome in neuroblastoma to make subsequent curative multimodal treatment possible.

## Author contributions

**Conceptualization:** Holger Lode.

**Data curation:** Holger Lode, Nikolai Siebert, Karoline Ehlert, Winfried Barthlen.

**Formal analysis:** Nikolai Siebert, Karoline Ehlert.

**Investigation:** Holger Lode.

**Methodology:** Karoline Ehlert.

**Supervision:** Holger Lode, Winfried Barthlen.

**Writing – original draft:** Holger Lode.

**Writing – review & editing:** Günter Henze.

Holger Lode orcid: 0000-0002-1201-208X.
